# Trends in mortality involving chronic obstructive pulmonary disease and obesity in the United States, 1999–2023: A population-based observational study

**DOI:** 10.1097/MD.0000000000050980

**Published:** 2026-10-02

**Authors:** Ammaar Ali Khan, Syed Azfar Hussain, Zainab Binte Ahmad, Raqib Ali, Hasibullah Aminpoor, Laiba Hanif, Muhammad Sameer Khan, Arshalooz Mahfooz, Muhammad Salik Uddin, Shaheer Qureshi

**Affiliations:** a School of Medicine, Dow University of Health Sciences, Karachi, Pakistan; b Faculty of Medicine, Kabul University of Medical Sciences “Abu Ali Ibn Sina”, Kabul, Afghanistan; c Department of Internal Medicine, Resident Physician, Rabia Balkhi National Complex Hospital, Kabul, Afghanistan.

**Keywords:** age-adjusted mortality, CDC WONDER, chronic obstructive pulmonary disease, health disparities, mortality trends, obesity, temporal trends

## Abstract

Chronic obstructive pulmonary disease (COPD) and obesity are major US public health conditions that frequently coexist. Despite adverse respiratory and cardiometabolic effects of obesity, an apparent “obesity paradox,” whereby obesity has been associated with lower mortality in some patients with COPD, has been described. National mortality trends involving concurrent COPD and obesity remain poorly characterized. We examined temporal, demographic, and geographic patterns from 1999 to 2023. We conducted a retrospective, population-based analysis of the Centers for Disease Control and Prevention Wide-ranging Online Data for Epidemiologic Research Multiple Cause of Death database. Deaths among adults aged ≥25 years listing COPD (International Classification of Diseases, 10th Revision: J40–J44) and obesity (International Classification of Diseases, 10th Revision: E66) were identified. Age-adjusted mortality rates per 100,000 were calculated using direct standardization to the 2000 US population. Joinpoint Regression Program version 5.3.0.0 estimated annual percent changes (APCs) and average annual percent changes with 95% confidence intervals (CIs). Analyses were stratified by age, sex, race/ethnicity, region, urbanization, and state. Among 1,30,958 deaths, the age-adjusted mortality rate increased from 0.78 (95% CI: 0.74–0.82) in 1999 to 2.63 (95% CI: 2.57–2.68) in 2023 (average annual percent change: +5.14%; 95% CI: 4.09–6.21; *P* < .001). After increasing significantly through 2013, mortality increased nonsignificantly during 2013 to 2018 (APC: +2.11%; 95% CI: −0.39 to 4.67), rose markedly during 2018 to 2021 (APC: +17.45%; 95% CI: 10.18–25.19), and declined significantly through 2023 (APC: −13.47%; 95% CI: −18.32 to −8.34). Males and adults aged ≥65 years had higher mortality burdens, with variation across racial/ethnic groups, urbanization levels, regions, and states. Interpretation is limited by unavailable individual-level factors, including smoking, COPD severity, comorbidities, and socioeconomic characteristics. Mortality involving concurrent COPD and obesity increased substantially from 1999 to 2023, with marked demographic and geographic variation and an increase during the COVID-19 pandemic. These population-level associations do not establish causality. Integrated pulmonary and metabolic care, targeted prevention, and improved access in underserved communities may help address observed disparities.

## Graphical abstract

**Figure:**
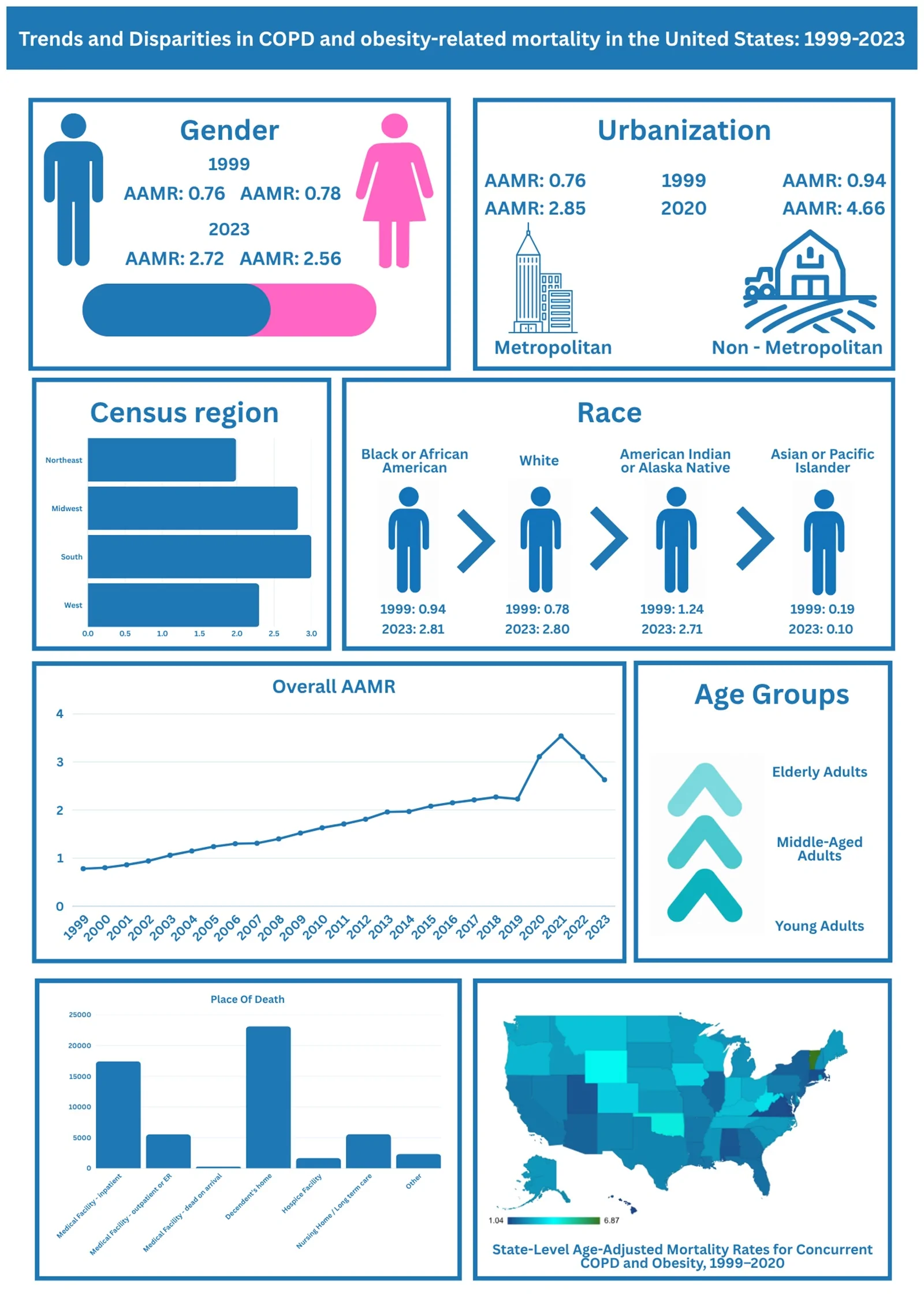

## 1. Introduction

Chronic obstructive pulmonary disease (COPD), a progressive disorder characterized by persistent airflow limitation and chronic respiratory symptoms, remains a major cause of morbidity and mortality in the United States.^[1]^ COPD accounts for a substantial proportion of deaths classified under chronic lower respiratory diseases, which remain among the leading causes of death nationally.^[2]^ More than 14 million US adults have been diagnosed with COPD, and its economic burden remains substantial, with annual healthcare costs exceeding $36 billion and projected to increase further.^[1,3]^ Obesity, defined as excess adiposity that impairs health, has also risen sharply over recent decades.^[4]^ According to nationally representative US survey data, 40.3% of adults had obesity during August 2021 to August 2023,^[5]^ while obesity-related medical expenditures were estimated at 260.6 billion dollars in 2016.^[6]^

The global burden of both COPD and obesity continues to intensify due to a mix of modifiable and non-modifiable factors shaped by biological, social and environmental conditions. These conditions frequently coexist, although prevalence estimates vary across populations and study settings, with obesity reported in approximately 35% to 54% of individuals with COPD,^[7]^ potentially reflecting shared factors such as reduced physical activity, metabolic dysfunction, and systemic inflammation.^[8,9]^ Although obesity impairs lung mechanics, it has been associated with lower mortality among individuals with COPD, a pattern often referred to as the “obesity paradox.”^[10]^ Proposed explanations include greater metabolic and energy reserves during chronic illness and differences in body composition, while methodological factors such as confounding by smoking, disease severity, reverse causation, and the limitations of body mass index as a measure of adiposity may also contribute to this apparent association.

However, despite these observations, population-level trends in mortality involving both conditions remain unexplored. Existing evidence is often limited by narrow cohorts, short follow-up or restricted demographic assessment, which limits population-level insight. Using Centers for Disease Control and Prevention’s Wide-ranging Online Data for Epidemiologic Research (CDC WONDER) data from 1999 through 2023, we examined mortality among US adults aged 25 years and older with COPD and concurrent obesity. Given established demographic and geographic heterogeneity in both COPD and obesity burden, we examined mortality across age, sex, race/ethnicity, state, census region, and urbanization level to characterize disparities in the joint mortality burden. This analysis aims to identify high risk populations while clarifying demographic and regional disparities to support targeted interventions and reduce preventable mortality.

## 2. Materials and methods

### 2.1. Population and study setting

This study utilizes a retrospective descriptive analysis design, examining data from the CDC WONDER database.^[11]^ The study utilized codes from the International Statistical Classification of Diseases and Related Health Problems, 10th Revision (ICD-10) data were extracted using the Multiple Cause of Death files.

The study population includes individuals aged 25 years and older. Obesity-related deaths were identified using ICD-10 code E66, including obesity due to excess calories (E66.0), drug-induced obesity (E66.1), extreme obesity with alveolar hypoventilation (E66.2), other obesity (E66.8), and unspecified obesity (E66.9). COPD-related deaths were identified using ICD-10 codes J40–J44, encompassing bronchitis not specified as acute or chronic (J40), chronic bronchitis (J41–J42), emphysema (J43), and other COPD (J44). Deaths were included when both obesity and COPD were recorded among the multiple causes of death.^[12]^ The objective is to analyze trends in mortality associated with obesity and COPD across the United States from 1999 to 2023, with a particular focus on age-related and race-specific trends within these conditions.

This study used publicly available, de-identified, aggregate mortality data from the CDC WONDER database and did not involve interaction with human participants or access to identifiable private information. Therefore, institutional review board approval and informed consent were not required.

### 2.2. Data abstraction

Data were extracted on demographic characteristics (age, sex, race/ethnicity) and place of death (categorized as medical facilities, nursing or long-term care facilities, hospices, decedent’s homes, or other unclassified locations). The age groups considered include young adults (25–44 years), middle-aged adults (45–64 years), older adults (65 years and above). For racial/ethnic groups, data were extracted for Hispanic/Latino, non-Hispanic Black/African American, and non-Hispanic White populations. Non-Hispanic American Indian or Alaska Native and non-Hispanic Asian populations were excluded from race/ethnicity-specific trend analyses because annual estimates were extensively suppressed or designated as unreliable in CDC WONDER, precluding stable estimation of temporal trends. Geographic classification by urbanization was based on the 2013 National Center for Health Statistics Urban-Rural Classification Scheme for Counties, the most recent urbanization classification available within the CDC WONDER Multiple Cause of Death query interface used for this analysis. For the present analysis, counties classified as large central metro, large fringe metro, medium metro, or small metro were grouped as metropolitan, whereas micropolitan and noncore counties were grouped as nonmetropolitan. Additionally, the United States was segmented into 4 regions, namely Northeast, Midwest, South, and West based on the US Census Bureau’s classification. Individual-level clinical variables, including smoking history, COPD severity, body mass index, and detailed comorbidity profiles, were not available in the CDC WONDER mortality files and therefore could not be incorporated into the analysis.

### 2.3. Data analysis

The primary analysis involved examining age-adjusted mortality rates (AAMR) per 100,000 individuals, standardized to the 2000 US population.^[13]^ Temporal trends in mortality were analyzed using the Joinpoint Regression Program (Version 5.3.0.0, National Cancer Institute).^[14]^ Log-linear regression models were fitted to identify changes in temporal trends. Model selection was performed using the permutation test with 4499 permutations and an overall significance level of 0.05. The number of joinpoints was permitted to range from 0 to 4, with a minimum of 2 observations between adjacent joinpoints and at least 2 observations between a joinpoint and either end of the data series. Annual percent changes (APCs) were estimated for individual trend segments, while average annual percent changes (AAPCs) were calculated across the entire study period. 95% confidence intervals (CIs) for APCs and AAPCs were calculated using the parametric method. Trends were considered statistically significant when *P* < .05.

## 3. Results

### 3.1. Overall mortality trends

A total of 1,30,958 deaths related to COPD and obesity were recorded among US adults aged ≥25 years from 1999 to 2023. The overall AAMR increased significantly from 0.78 (95% CI: 0.74 to 0.82) in 1999 to 2.63 (95% CI: 2.57 to 2.68) in 2023, with an AAPC of +5.14% (95% CI: 4.09 to 6.21, *P* < .000001). The AAMR rose significantly from 1999 to 2013, reaching1.96 (APC: +6.69%, 95% CI: 6.15 to 7.23, *P* < .000001), followed by a slower, nonsignificant increase to 2.27 in 2018 (APC: +2.11%, 95% CI: −0.39 to 4.67, *P* = .0928). A steep and significant increase occurred until 2021, peaking at an AAMR of 3.54 (APC: +17.45%, 95% CI: 10.18 to 25.19, *P* < .000093), followed by a significant decrease from 2021 to 2023 (APC: −13.47%, 95% CI: −18.32 to −8.34, *P* < .000096; Fig. 1, Table S1, Supplemental Digital Content 1).

**Figure 1.**
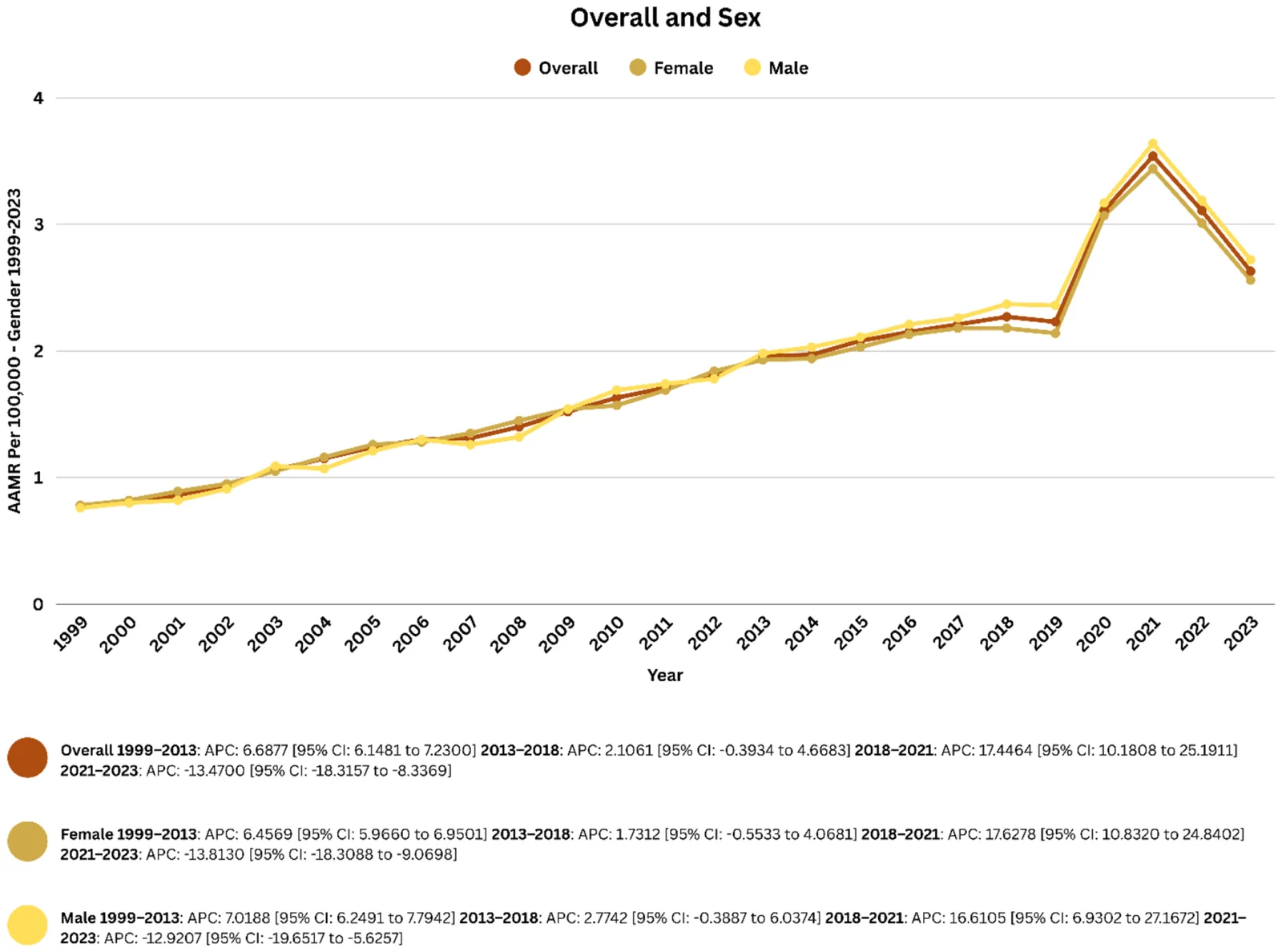
Overall and sex-stratified age-adjusted mortality rates (AAMRs) for concurrent chronic obstructive pulmonary disease (COPD) and obesity in the United States, 1999–2023. AAMR = age-adjusted mortality rate, APC = annual percent change, CI = confidence interval, COPD = chronic obstructive pulmonary disease.

### 3.2. Mortality trends by sex

Among females (68,881 deaths; 52.6% of the total), the AAMR increased significantly from 0.78 in 1999 to 1.93 in 2013 (APC: +6.46%, 95%, CI: 5.97 to 6.95, *P* < .000001), followed by a nonsignificant modest increase to 2.18 in 2018 (APC: +1.73%, 95% CI: −0.55 to 4.07, *P* = .127), and a significant surge to 3.44 in 2021 (APC: +17.63%, 95% CI: 10.83 to 24.84, *P* < .000042), before declining significantly to 2.56 in 2023 (APC: −13.81%, 95%, CI: −18.31 to −9.07, *P* < .000035). Males accounted for 62,077 deaths (47.4% of the total) and had higher AAMRs than females throughout. The AAMR rose significantly from 0.76 in 1999 to 1.98 in 2013 (APC: +7.02%, 95%, CI: 6.25 to 7.79, *P* < .000001), then increased nonsignificantly to 2.37 in 2018 (APC: +2.77%, 95%, CI: −0.39 to 6.04, *P* = .0814). A significant escalation was observed from 2018 to 2021, peaking at 3.64 (APC: +16.61%, 95%, CI: 6.93 to 27.17, *P* = .00194), followed by a pronounced and significant decrease to 2.72 in 2023 (APC: −12.92%, 95%, CI: −19.65 to −5.63, *P* = .00243; Fig. 1, Table S1, Supplemental Digital Content 1).

### 3.3. Mortality trends by race/ethnicity

For the non-Hispanic White population, the AAMR increased significantly and steadily from 0.82 in 1999 to 2.24 in 2014 (APC: +7.06%, 95%, CI: 6.68 to 7.44, *P* < .000001), followed by no significant temporal change between 2014 and 2018 (APC: +1.18%, 95%, CI: −1.57 to 4.01, *P* = .377), surged significantly to a peak of 4.04 in 2021 (APC: +17.66%, 95%, CI: 11.68 to 23.96, *P* < .000010), and then declined significantly to 3.11 in 2023 (APC: −11.21%, 95%, CI: −15.85 to −6.31, *P* < .000311). Among non-Hispanic Black or African American populations, mortality rose significantly from 0.98 in 1999 to 2.53 in 2018 (APC: +5.22%, 95%, CI: 4.51 to 5.93, *P* < .000001), followed by a sharp, significant increase to 4.15 in 2021 (APC: +19.67%, 95%, CI: 3.96 to 37.76, *P* = .0154), and a marked, significant decline to 2.89 in 2023 (APC: −18.03%, 95%, CI: −27.94 to −6.75, *P* = .00467). For Hispanic or Latino populations, the AAMR increased significantly and gradually from 0.45 in 1999 to 1.48 in 2021 (APC: +6.19%, 95%, CI: 4.95 to 7.45, *P* < .000001), followed by a nonsignificant decline to 0.90 in 2023 (APC: −17.60%, 95%, CI: −38.28 to 10.00, *P* = .177). The AAPC for 1999 to 2023 was highest for non-Hispanic Whites (+5.65%, *P* < .000001), followed by non-Hispanic Blacks (+4.72%, *P* = .000005), and Hispanic or Latinos (+3.79%, *P* = .021; Fig. 2, Table S2, Supplemental Digital Content 2).

**Figure 2.**
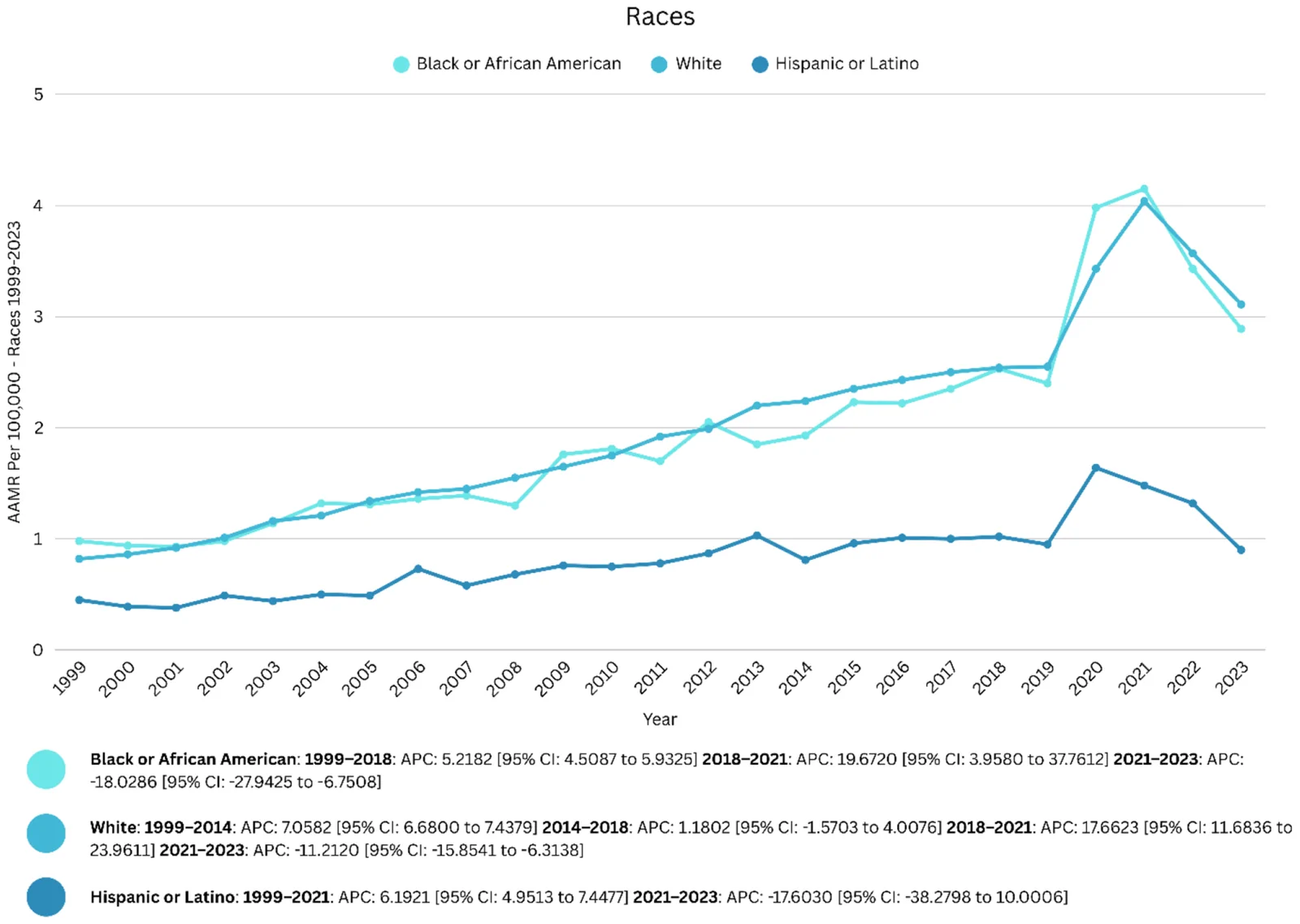
Age-adjusted mortality rates (AAMRs) stratified by race/ethnicity for concurrent COPD and obesity in the United States, 1999–2023. AAMR = age-adjusted mortality rate, APC = annual percent change, CI = confidence interval, COPD = chronic obstructive pulmonary disease.

### 3.4. Age-specific mortality trends

For older adults (≥65 years), the AAMR followed a significant and consistent upward trajectory from an AAMR of 2.53 in 1999 to 7.47 in 2018 (APC: +5.70%, 95% CI: 5.33 to 6.07, *P* < .000001), followed by a significant and dramatic escalation from 2018 to 2021, peaking at 11.95 (APC: +15.45%, 95% CI: 7.48 to 24.02, *P* = .000558), and a sharp, significant decline through 2023 to a final rate of 9.23 (APC: −11.11%, 95% CI: −16.74 to −5.09, *P* = .00145). For middle-aged adults (45–64 years), the AAMR rose significantly and steadily from 1.17 in 1999 to 2.94 in 2014 (APC: +6.37%, 95% CI: 5.83 to 6.90, *P* < .000001), grew nonsignificantly to 3.34 in 2018 (APC: +1.15%, 95% CI: −2.96 to 5.42, *P* = .564), surged significantly from 2018 to 2021 to a rate of 5.06 (APC: +16.28%, 95% CI: 8.02 to 25.17, *P* = .000617), and was followed by a significant decline through 2023 to a final rate of 3.61 (APC: −15.11%, 95% CI: −20.96 to −8.84, *P* < .000222). For young adults (25–44 years), the AAMR rose significantly from 0.11 in 1999 to 0.32 in 2012 (APC: +8.66%, 95% CI: 6.58 to 10.79, *P* < .000001), followed by a nonsignificant decline through 2018 (APC: −3.19%, 95% CI: −8.95 to 2.93, *P* = .276). The AAMR subsequently increased to 0.48 in 2021, although this trend was nonsignificant and estimated with a wide CI (APC: +15.66%, 95% CI: −15.12 to 57.61, *P* = .330), followed by a nonsignificant decline between 2021 and 2023 (APC: −16.65%, 95% CI: −36.50 to 9.40, *P* = .173). The wide CIs surrounding these latter estimates indicate substantial uncertainty in the observed trends (Fig. 3, Table S3, Supplemental Digital Content 3).

**Figure 3.**
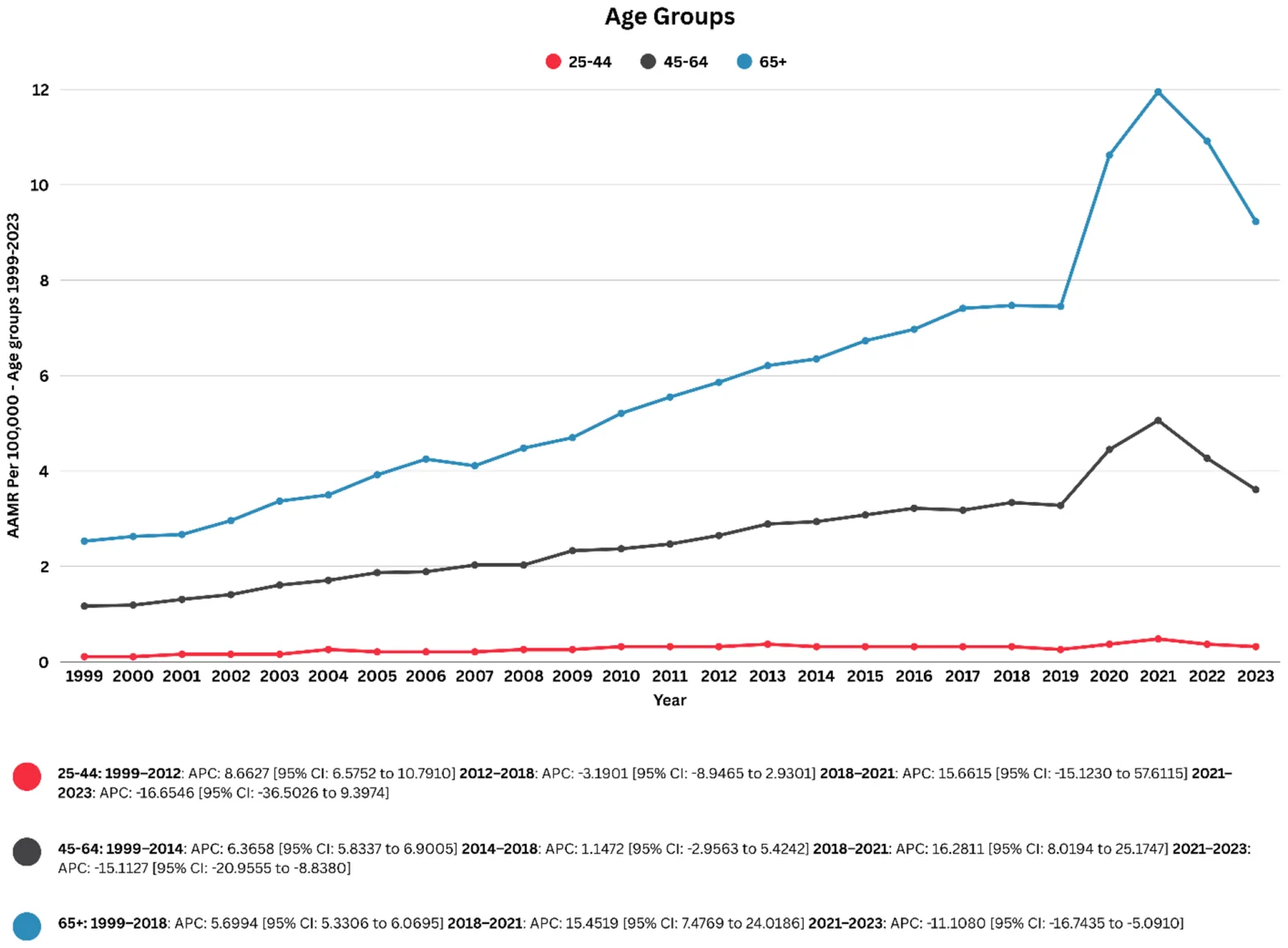
Age-adjusted mortality rates (AAMRs) stratified by age group for concurrent COPD and obesity in the United States, 1999–2023. AAMR = age-adjusted mortality rate, APC = annual percent change, CI = confidence interval, COPD = chronic obstructive pulmonary disease.

### 3.5. Geographic disparities

For metropolitan areas, the AAMR increased significantly from 0.76 in 1999 to 1.91 in 2015 (APC: +6.14%, 95% CI: 5.82 to 6.46, *P* < .000001), followed by a period of no significant temporal change to 2018 (APC: −0.51%, 95% CI: −6.07 to 5.38, *P* = .851), and a significant and abrupt rise to 2.85 in 2020 (APC: +18.63%, 95% CI: 12.90 to 24.66, *P* < .000003). Nonmetropolitan areas had a significant and rapid escalation from 0.94 in 1999 to 2.56 in 2012 (APC: +8.05%, 95% CI: 7.18 to 8.94, *P* < .000001), followed by a significant and moderate uptrend to 3.32 in 2018 (APC: +3.89%, 95% CI: 1.46 to 6.38, *P* = .00385), and a significant and dramatic spike to 4.66 in 2020 (APC: +16.81%, 95% CI: 6.43 to 28.20, *P* = .00300; Fig. 4, Table S4, Supplemental Digital Content 4).

**Figure 4.**
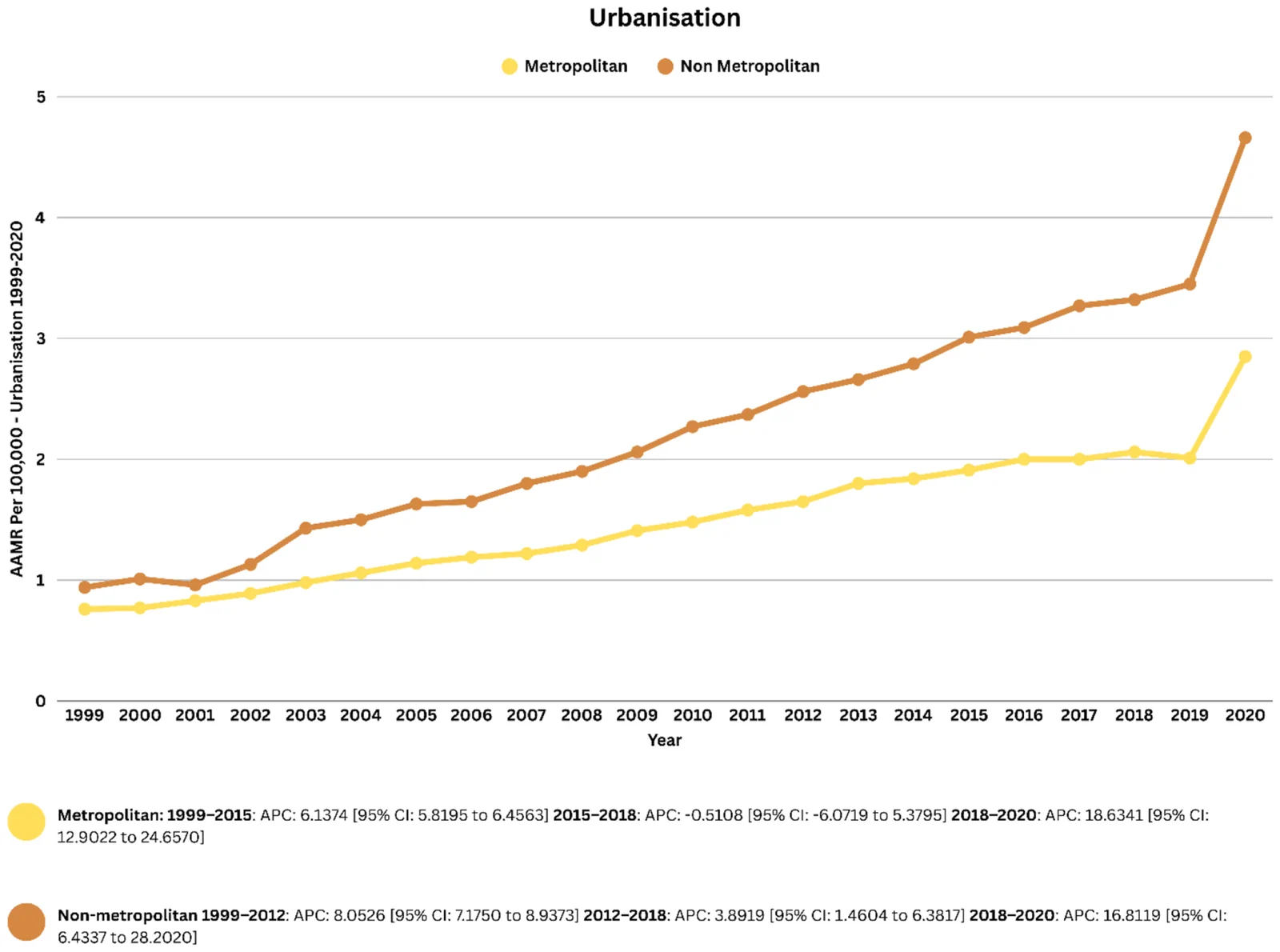
Age-adjusted mortality rates (AAMRs) Stratified by urbanization level for concurrent COPD and obesity in the United States, 1999–2023. AAMR = age-adjusted mortality rate, APC = annual percent change, CI = confidence interval, COPD = chronic obstructive pulmonary disease.

In the Northeast, the AAMR increased significantly from 0.70 in 1999 to 1.84 in 2018 (APC: +5.33%, 95% CI: 4.89 to 5.77, *P* < .000001), rose significantly and rapidly to 2.57 in 2021 (APC: +10.68%, 95% CI: 0.32 to 22.12, *P* = .0438), and then decreased significantly to 1.99 in 2023 (APC: −12.55%, 95% CI: −20.67 to −3.60, *P* = .00991). For the West region, the AAMR rose significantly from 0.92 in 1999 to 2.23 in 2018 (APC: +4.34%, 95% CI: 3.83 to 4.86, *P* < .000001), increased nonsignificantly to 3.24 in 2021 (APC: +11.86%, 95% CI: -0.045 to 25.69, *P* = .0584), and was followed by a significant fall to 2.30 in 2023 (APC: −13.35%, 95% CI: −21.46 to −4.41, *P* = .00683). The South showed a significant surge in AAMR from 0.72 in 1999 to 1.96 in 2013 (APC: +7.39%, 95% CI: 6.69 to 8.10, *P* < .000001), a nonsignificant increase to 2.26 in 2018 (APC: +2.00%, 95% CI: −0.75 to 4.84, *P* = .143), a marked and significant escalation to 3.86 in 2021 (APC: +20.97%, 95% CI: 12.37 to 30.24, *P* = .000073), followed by a significant reduction to 3.00 in 2023 (APC: −11.24%, 95% CI: −17.31 to −4.73, *P* = .00282). The Midwest showed a significant increase in AAMR from 0.85 in 1999 to 2.49 in 2015 (APC: +7.05%, 95% CI: 6.37 to 7.75, *P* < .000001), followed by no significant temporal change through 2018 (APC: −0.43%, 95% CI: −11.51 to 12.05, *P* = .939), followed by a steep and significant rise to 4.08 in 2021 (APC: +17.08%, 95% CI: 5.08 to 30.43, *P* = .00739), and a subsequent significant decrease to 2.82 in 2023 (APC: −17.04%, 95% CI: −24.99 to −8.24, *P* = .00138; Fig. 5, Table S5, Supplemental Digital Content 5).

**Figure 5.**
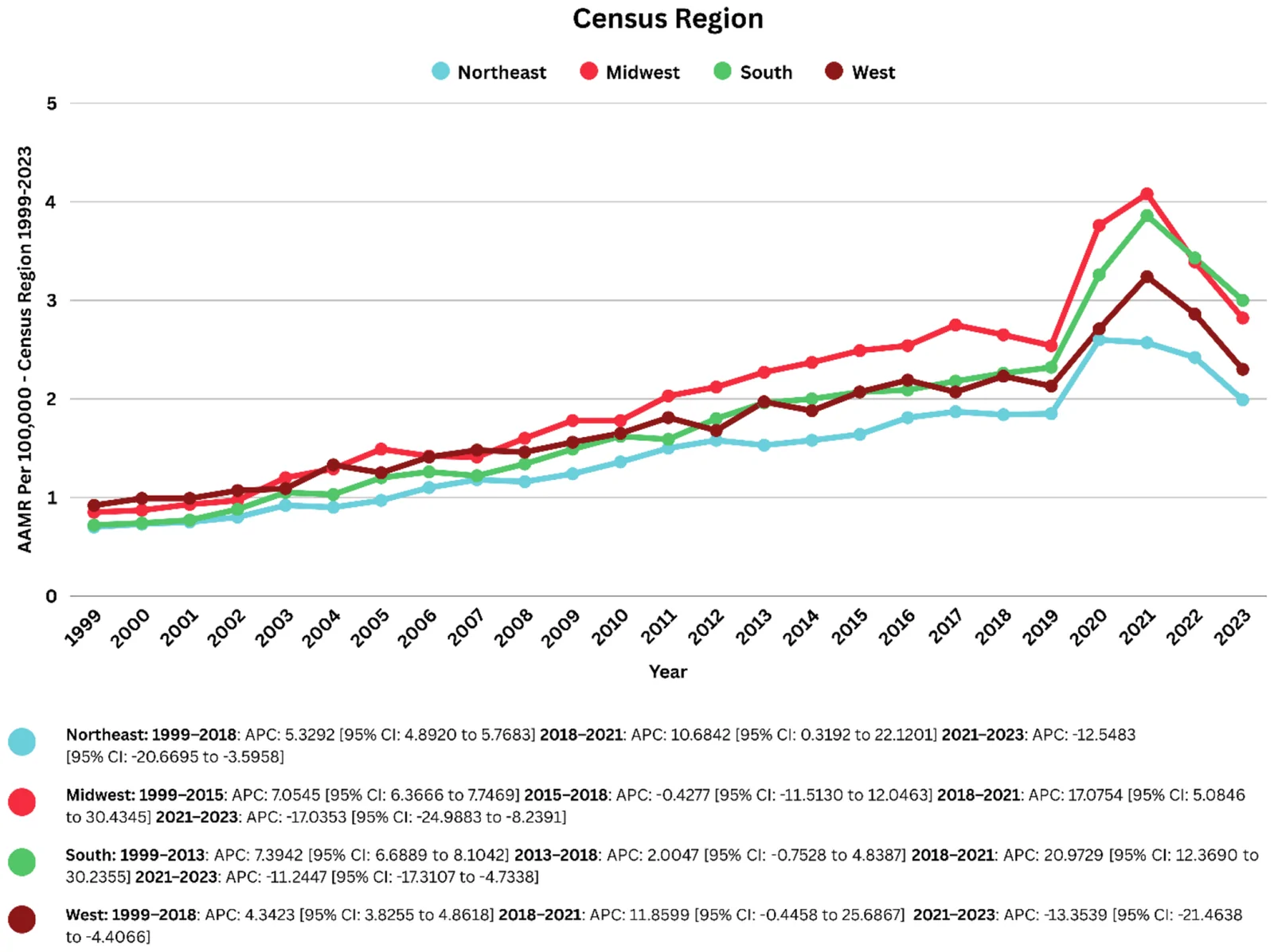
Age-adjusted mortality rates (AAMRs) stratified by census region for concurrent COPD and obesity in the United States, 1999–2023. AAMR = age-adjusted mortality rate, APC = annual percent change, CI = confidence interval, COPD = chronic obstructive pulmonary disease.

At the state level, mortality was examined separately for 1999 to 2020 and 2021 to 2023 because estimates for these periods were derived from distinct CDC WONDER files that use different population denominators and, therefore, were not pooled. During 1999 to 2020, substantial geographic variation was observed, with the highest cumulative AAMR in Vermont (6.87), followed by Oklahoma (4.39), Wyoming (4.11), West Virginia (3.45), and Iowa (3.25), while Hawaii had the lowest AAMR (1.04). During 2021 to 2023, the highest AAMR was observed in Oklahoma (13.63), followed by Vermont (11.75), South Carolina (9.72), Wyoming (8.97), and Rhode Island (8.09), while Hawaii again had the lowest AAMR (1.45). State-specific estimates for both periods are presented in Table S7, Supplemental Digital Content 6 and mapped in Figure 6.

**Figure 6.**
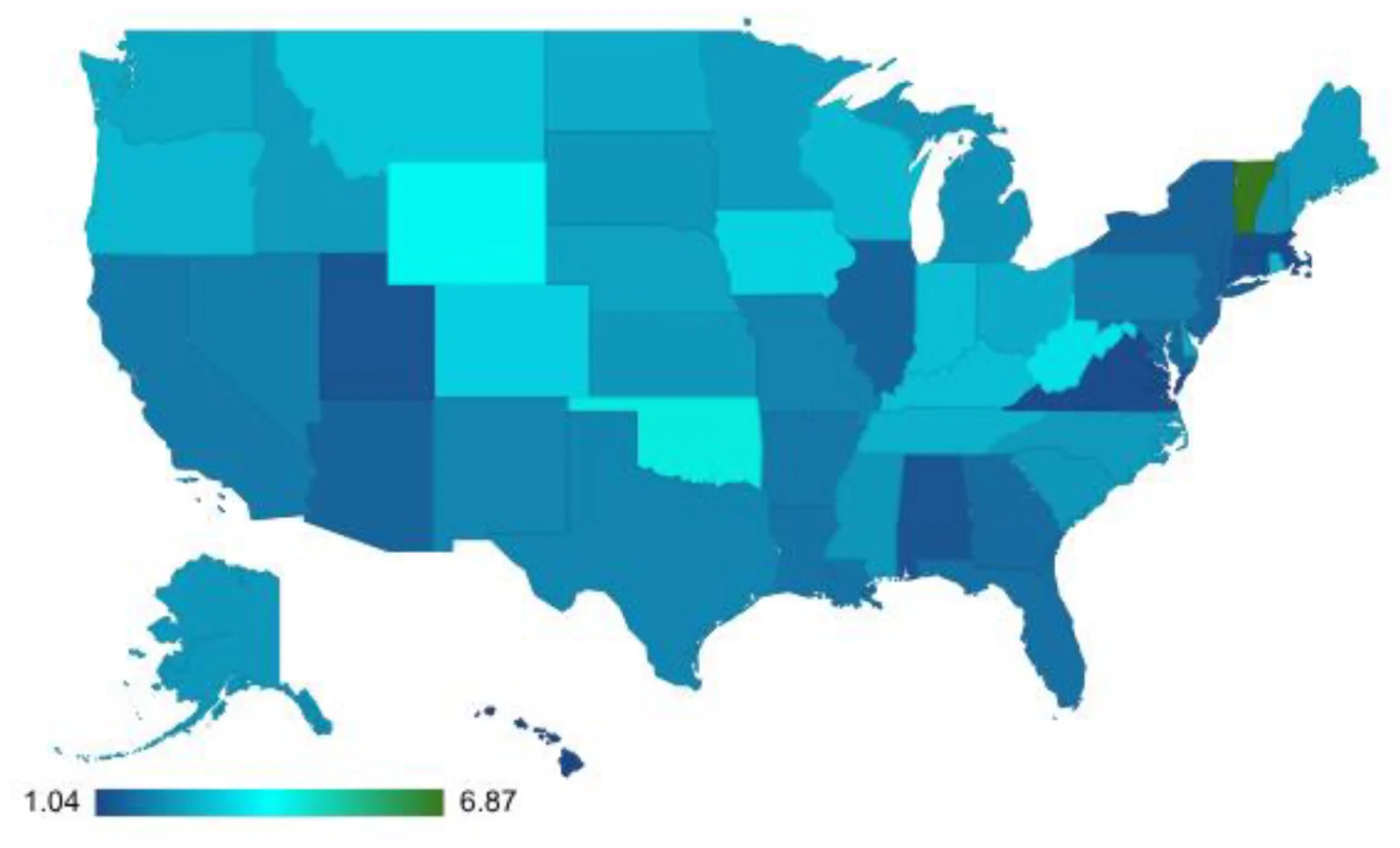
State-level age-adjusted mortality rates (AAMRs) for concurrent COPD and obesity in the United States, 1999 to 2020. Cumulative state-level AAMRs per 100,000 population among adults aged ≥25 years. Rates standardized to the 2000 US population. AAMR = age-adjusted mortality rate, APC = annual percent change, COPD = chronic obstructive pulmonary disease.

### 3.6. Place of death

Analysis of mortality data by location revealed that the decedent’s home was the most common place of death, accounting for 37.39% of cases (48,971 deaths). This was followed by inpatient medical facilities, which accounted for 33.78% of deaths (44,237 deaths). A smaller but notable proportion of deaths occurred in outpatient or emergency room settings (11.03%, 14,446 deaths) and nursing homes or long-term care facilities (10.61%, 13,899 deaths). Other locations accounted for 3.76% of deaths (4927 deaths), followed by hospice facilities (2.38%, 3123 deaths), and those declared dead on arrival (0.87%, 1136 deaths). The place of death was unknown in 0.14% of cases (182 deaths), while 0.03% (37 deaths) had unknown medical-facility status. (Table S6, Supplemental Digital Content 7).

## 4. Discussion

Our analysis of US mortality from 1999 to 2023 reveals that the combination of COPD and obesity has become a significant public health crisis, with AAMRs for patients with both conditions more than tripling. This long-term trend was catastrophically accelerated by the COVID-19 pandemic, though the resulting burden was distributed unevenly. Stark disparities appeared across demographic and geographic lines, with the highest mortality rate observed among Non-Hispanic White and Black populations, as well as residents of nonmetropolitan areas. While older adults predictably bore the greatest burden, alarming mortality increases were also evident among middle-aged individuals, signaling a rise in premature death. This discussion therefore examines the drivers of these trends and inequities to clarify why this convergence has had such a devastating impact on vulnerable populations.

In patients with COPD, coexisting obesity may contribute to respiratory impairment through interacting inflammatory and mechanical mechanisms. Obesity is associated with systemic inflammation, including increased production of pro-inflammatory mediators such as TNF-α and IL-6.^[15,16]^ Importantly, COPD-specific studies indicate that obesity is associated with altered systemic inflammatory and immune profiles, although the direction and magnitude of these changes may vary according to disease severity and patient phenotype.^[17]^ Simultaneously, central obesity restricts diaphragmatic excursion and reduces chest wall compliance, shrinking lung volumes and increasing the work of breathing.^[18]^ In patients with coexisting COPD and obesity, these inflammatory and mechanical effects may therefore interact to reduce respiratory reserve and increase vulnerability to adverse respiratory outcomes.^[19]^

The pre-pandemic increase in mortality likely reflects several concurrent public health and clinical factors rather than a single identifiable cause. Rising obesity prevalence in the US may have contributed by increasing the population with metabolic dysfunction and obesity-related respiratory impairment.^[20]^ The growing burden of associated conditions, including type 2 diabetes and cardiovascular disease, may have further increased vulnerability among individuals with COPD.^[21,22]^ Concurrently, improvements in COPD management and survival have resulted in a larger aging population living with chronic respiratory disease, in whom comorbidities and frailty may accumulate over time.^[23]^ Temporal changes in death-certificate coding and recognition of obesity as a contributing cause of death may also have influenced the observed mortality trends.^[24]^ Importantly, the present data do not permit quantification of the relative contributions of changes in disease prevalence, population characteristics, and coding practices to the pre-pandemic increase. These preexisting vulnerabilities were subsequently compounded during the COVID-19 pandemic by the direct and indirect effects of the pandemic, including disruptions in chronic disease care.^[25,26]^

While the overall mortality trajectories for men and women were similar, men consistently exhibited higher AAMRs, a disparity that is likely multifactorial and may reflect biological, behavioral, and exposure-related differences. Sex-related differences in body composition and fat distribution may contribute, as men generally exhibit greater visceral adiposity; however, these patterns vary with age, hormonal status, and race/ethnicity and therefore cannot uniformly explain the observed mortality difference.^[27,28]^ Visceral adiposity has been associated with greater systemic inflammatory activity, which may be particularly relevant in the setting of COPD.^[29]^ Women, in contrast, may benefit from the cardioprotective and anti-inflammatory effects of estrogen, which can potentially buffer some of the systemic damage from both COPD and obesity.^[30]^ These biological differences are likely amplified by historical gaps in risk exposure. Higher rates of smoking and greater lifetime occupational exposure to lung irritants among men in previous decades mean that older male cohorts may carry a greater cumulative burden of lung damage.^[31]^ Finally, these factors are compounded by well-documented gender differences in healthcare engagement. Men’s tendency to delay seeking medical care can lead to later diagnoses and less consistent management of their chronic conditions, allowing the diseases to progress to more severe and ultimately fatal stages.^[32]^ Taken together, the observed sex disparity is unlikely to reflect differences in adiposity alone and should instead be interpreted in the context of interacting biological, behavioral, and exposure-related factors.

Furthermore, our analysis also reveals that race and ethnicity are powerful predictors of mortality from this disease combination, reflecting deep and persistent health inequities. The non-Hispanic Black population has consistently shouldered the heaviest burden, experiencing the highest death rates and the most severe mortality peak during the pandemic. This tragic disparity is linked to a higher prevalence of underlying comorbidities like hypertension and diabetes that is eventually compounded by the effects of systemic factors like residential segregation and greater exposure to environmental air pollution, which directly exacerbates lung disease.^[32,33]^ For the non-Hispanic White population, the story is one of rapid escalation. This group had the fastest overall rate of increasing mortality, a trend likely fueled by the collision of the obesity epidemic with the long-term consequences of historically high smoking rates, particularly in the hard-hit geographic regions of the rural South and Midwest.^[34]^ In contrast, the Hispanic population had significantly lower mortality rates throughout the study period. This finding may be reflective of the “Hispanic Paradox, a phenomenon often attributed to protective factors like strong social support systems and the “healthy immigrant effect.”^[35]^ However, it is crucial to note that their mortality rate still showed a significant upward trend, indicating this group is by no means immune to the growing threat.

Our analysis of mortality by age reveals 2 distinct narratives: a massive and expected burden in older adults and a deeply concerning surge in premature death among the middle-aged. The high mortality rates in adults aged 65 and over are tragically predictable. This reflects the combined impact of immunosenescence, the natural aging of the immune system, and the accumulation of comorbidities, which together drastically reduce a person’s physiological reserve to survive a severe respiratory illness.^[36]^ The more alarming story is the steep rise in mortality among middle-aged adults (45–64 years). The rise in premature mortality among middle-aged adults (45–64 years) is consistent with evidence that obesity substantially advances the timing of death. A study using the NHANES I–III data demonstrated that, among healthy nonsmoking adults aged 21 to 55 years, obesity (BMI ≥ 30 kg/m^2^) was associated with mortality occurring approximately 9.4 years earlier, with severe obesity (BMI ≥ 35 kg/m^2^) advancing death by more than 14 years. Obesity accelerates the development of major cardiovascular risk –factors – including hypertension, type 2 diabetes, and dyslipidemia – which promote earlier atherosclerosis and cardiac dysfunction. As obesity prevalence has risen over recent decades, prolonged exposure to these risk factors likely shifts cardiovascular events and death into midlife, contributing to the observed increase in premature mortality.^[37,38]^ While younger adults saw smaller increases that weren’t statistically significant, likely reflecting their greater physiological resilience, the data clearly show that a risk once largely confined to old age has now become a major factor of mortality in the middle-aged.

The most profound geographic disparity in our findings is the widening mortality chasm between metropolitan and nonmetropolitan America. The alarmingly high death rates in rural areas are the result of a “vicious cycle” of disadvantage. These communities simultaneously face a higher prevalence of the primary risk factors (obesity and smoking), a decaying healthcare infrastructure with fewer specialists to manage complex chronic disease, and greater socioeconomic challenges like poverty.^[39-41]^ In contrast, urban centers benefit from a “virtuous cycle” of concentrated resources, including better access to specialized medical care and public health programs that support chronic disease management. This fundamental divide in resources and risk factors is the primary driver of the geographic patterns observed in our study.

The mortality differences between census regions are largely an expression of this underlying urban-rural dynamic. The disproportionately high death rates in the South and Midwest are not a regional quirk but a direct reflection of the vast, medically underserved rural populations within those states, which often overlap with known “hotspots for chronic conditions like the “Diabetes Belt.”^[42]^ Conversely, the lower overall rates in the more urbanized Northeast and West are skewed by large metropolitan areas with robust health systems. At the state level, the highest mortality rates were seen in states like Oklahoma, West Virginia, and Vermont, all of which are characterized by significant rural populations, high rates of poverty, and a heavy burden of the very risk factors driving this lethal combination of diseases. These geographic patterns should nevertheless be interpreted in light of the resolution of CDC WONDER data. Aggregation at the state and broad urbanization levels may obscure substantial within-state and within-county heterogeneity, while suppression of sparse counts can further limit characterization of mortality in smaller geographic populations. Consequently, localized disparities may be greater or differ from those apparent in the aggregated estimates presented here.

The setting in which these deaths occurred provides additional context regarding end-of-life patterns but should be interpreted cautiously. Home was the most frequently recorded place of death (37.9%), followed by inpatient medical facilities (33.78%). Although geographic barriers and distance from emergency care may contribute to home death in some populations,^[43]^ place of death may also reflect patient and family preferences, advance care planning, hospice availability, cultural factors, socioeconomic circumstances, and differences in disease trajectory. Similarly, deaths occurring in outpatient or emergency room settings (11.03%) may reflect acute clinical deterioration as well as differences in access to and utilization of primary and preventive care.^[44]^ Deaths in nursing homes or long-term care facilities (10.61%) may be influenced by age, functional status, comorbidity burden, socioeconomic circumstances, and the availability and quality of institutional or palliative care.^[45]^ Importantly, CDC WONDER does not provide information on individual end-of-life preferences, care trajectories, hospice utilization, or the circumstances surrounding death; therefore, the observed place-of-death patterns cannot be attributed directly to healthcare access barriers or systemic disadvantage.

These findings demand a multi-pronged response. Clinicians must adopt aggressive, integrated management of COPD and obesity as a unified high-risk condition, particularly in middle-aged adults, alongside proactive care planning.^[46]^ Policymakers must address the urban-rural divide through investment in rural infrastructure, specialist incentives, and telehealth expansion.^[47]^ Policymakers should also address structural contributors to racial and geographic disparities through strengthened air-quality and occupational exposure protections, targeted pollution-reduction strategies in disproportionately exposed communities, improved access to preventive pulmonary and metabolic care, and investment in underserved communities.^[48]^ Researchers should shift from description to intervention, identifying geographic and racial hotspots and testing integrated strategies addressing metabolic, pulmonary, and social determinants simultaneously.^[49]^

## 5. Limitations

This study’s findings are subject to several limitations. First, our analysis depends on the accurate coding of both COPD and obesity as contributing causes of death. Obesity, in particular, is substantially underreported on death certificates; a prior audit found that 96% of selected natural deaths were likely underreported as being associated with obesity.^[50]^ Consequently, reliance on death-certificate coding may lead to underestimation of the mortality burden involving concurrent COPD and obesity. Second, CDC WONDER lacks granular individual-level clinical data, including COPD severity, smoking history, body mass index, treatment patterns, and specific comorbidities. The absence of these variables prevents adjustment for important potential confounders; for example, temporal or demographic differences in smoking exposure, COPD severity, or comorbidity burden could contribute to observed mortality differences independently of obesity. Consequently, the observed trends cannot establish the independent contribution of obesity to mortality among individuals with COPD. Furthermore, finer cross-stratification by race/ethnicity and age group resulted in small cell counts and extensive data suppression under CDC WONDER reliability criteria, precluding reliable assessment of intersectional disparities. Future studies using sufficiently granular individual-level data are needed to examine these relationships. The wide CIs surrounding temporal estimates among younger adults indicate substantial uncertainty in these trends, likely reflecting the smaller number of deaths in this age stratum. Thus, the nonsignificant findings should not be interpreted as evidence of stable mortality rates. The absence of individual-level clinical information also precludes causal inference, and the observed relationships should therefore be interpreted as associations. Finally, as a population-level analysis, these findings are subject to ecological fallacy and cannot be used to infer individual-level risk. Future longitudinal cohort studies incorporating individual-level clinical, behavioral, socioeconomic, and treatment data are needed to validate these population-level associations, address residual confounding, and better characterize the relationship between concurrent COPD and obesity and mortality.

## 6. Conclusion

In conclusion, mortality involving concurrent COPD and obesity increased substantially among US adults from 1999 to 2023, with a marked increase during the COVID-19 pandemic and significant variation across demographic and geographic groups. Higher mortality rates were observed among men, older adults, and nonmetropolitan populations, with substantial differences also observed across racial/ethnic groups, census regions, and states. These findings identify populations experiencing a disproportionate mortality burden but, given the ecological nature of the analysis and limitations of death-certificate data, should not be interpreted as demonstrating causal relationships at the individual level. Future studies incorporating individual-level clinical, behavioral, socioeconomic, and healthcare-access data are needed to clarify the factors underlying these disparities and inform integrated approaches to COPD and obesity management. Public health efforts should prioritize measurable surveillance of disparities, including changes in AAMRs and demographic and geographic gaps over time, to evaluate progress and identify populations requiring targeted intervention.

## Author contributions

**Conceptualization:** Ammaar Ali Khan.

**Data curation:** Syed Azfar Hussain.

**Formal analysis:** Syed Azfar Hussain.

**Methodology:** Ammaar Ali Khan, Syed Azfar Hussain.

**Supervision:** Muhammad Salik Uddin, Shaheer Qureshi.

**Visualization:** Zainab Binte Ahmad.

**Writing – original draft:** Ammaar Ali Khan, Syed Azfar Hussain, Zainab Binte Ahmad, Raqib Ali, Hasibullah Aminpoor, Laiba Hanif, Muhammad Sameer Khan, Arshalooz Mahfooz.

**Writing – review & editing:** Ammaar Ali Khan, Zainab Binte Ahmad, Muhammad Salik Uddin, Shaheer Qureshi.














