# Supplementary material for: Trends in mortality involving chronic obstructive pulmonary disease and obesity in the United States, 1999–2023: A population-based observational study
Source: Medicine (Baltimore). 2026 Oct 2;105(40):e50980. doi: 10.1097/MD.0000000000050980 (PMC13632968; doi:10.1097/MD.0000000000050980)
Supplement: Supplementary file 1 [file medi-105-e50980-s001.docx]

Overall Age Adjusted Mortality Rate (95% CI)

| Year | Overall | Male | Female |
| --- | --- | --- | --- |
| 1999 | 0.78 (0.74–0.82) | 0.76 (0.71–0.82) | 0.78 (0.73–0.83) |
| 2000 | 0.80 (0.76–0.84) | 0.80 (0.74–0.86) | 0.82 (0.77–0.87) |
| 2001 | 0.86 (0.82–0.90) | 0.82 (0.76–0.88) | 0.89 (0.83–0.94) |
| 2002 | 0.94 (0.90–0.98) | 0.91 (0.85–0.96) | 0.95 (0.89–1.00) |
| 2003 | 1.06 (1.02–1.10) | 1.09 (1.02–1.15) | 1.05 (1.00–1.11) |
| 2004 | 1.15 (1.10–1.19) | 1.07 (1.01–1.14) | 1.16 (1.10–1.22) |
| 2005 | 1.24 (1.19–1.28) | 1.21 (1.14–1.27) | 1.26 (1.20–1.33) |
| 2006 | 1.30 (1.25–1.34) | 1.30 (1.23–1.37) | 1.28 (1.22–1.34) |
| 2007 | 1.31 (1.27–1.36) | 1.26 (1.20–1.33) | 1.35 (1.29–1.41) |
| 2008 | 1.40 (1.35–1.44) | 1.32 (1.25–1.38) | 1.45 (1.38–1.51) |
| 2009 | 1.52 (1.47–1.56) | 1.54 (1.47–1.61) | 1.54 (1.47–1.60) |
| 2010 | 1.63 (1.58–1.68) | 1.69 (1.61–1.76) | 1.57 (1.51–1.64) |
| 2011 | 1.71 (1.66–1.76) | 1.74 (1.66–1.81) | 1.69 (1.62–1.76) |
| 2012 | 1.81 (1.76–1.86) | 1.78 (1.71–1.86) | 1.84 (1.77–1.91) |
| 2013 | 1.96 (1.90–2.01) | 1.98 (1.90–2.06) | 1.93 (1.86–2.00) |
| 2014 | 1.97 (1.92–2.03) | 2.03 (1.96–2.11) | 1.94 (1.87–2.02) |
| 2015 | 2.08 (2.02–2.13) | 2.11 (2.03–2.19) | 2.03 (1.96–2.10) |
| 2016 | 2.15 (2.10–2.21) | 2.21 (2.13–2.29) | 2.13 (2.06–2.20) |
| 2017 | 2.21 (2.16–2.26) | 2.26 (2.18–2.34) | 2.18 (2.11–2.26) |
| 2018 | 2.27 (2.21–2.32) | 2.37 (2.29–2.45) | 2.18 (2.11–2.25) |
| 2019 | 2.23 (2.17–2.28) | 2.36 (2.28–2.44) | 2.14 (2.07–2.21) |
| 2020 | 3.11 (3.05–3.17) | 3.17 (3.08–3.26) | 3.07 (2.99–3.15) |
| 2021 | 3.54 (3.47–3.60) | 3.64 (3.55–3.74) | 3.44 (3.35–3.53) |
| 2022 | 3.11 (3.05–3.17) | 3.19 (3.10–3.28) | 3.01 (2.93–3.10) |
| 2023 | 2.63 (2.57–2.68) | 2.72 (2.63–2.80) | 2.56 (2.48–2.63) |

**Supplementary Table S1:** Overall Age Adjusted Mortality Rates (95% CI) stratified by year and sex
