# Supplementary material for: Trends in mortality involving chronic obstructive pulmonary disease and obesity in the United States, 1999–2023: A population-based observational study
Source: Medicine (Baltimore). 2026 Oct 2;105(40):e50980. doi: 10.1097/MD.0000000000050980 (PMC13632968; doi:10.1097/MD.0000000000050980)
Supplement: Supplementary file 2 [file medi-105-e50980-s002.docx]

| Overall Age Adjusted Mortality Rate (95% CI) | | |
| --- | --- | --- |
| Race | Year | Age-Adjusted Rate(95% CI) |
| Hispanic or Latino | 1999 | 0.45 (0.33–0.59) |
| Hispanic or Latino | 2000 | 0.39 (0.29–0.52) |
| Hispanic or Latino | 2001 | 0.38 (0.28–0.51) |
| Hispanic or Latino | 2002 | 0.49 (0.38–0.63) |
| Hispanic or Latino | 2003 | 0.44 (0.33–0.56) |
| Hispanic or Latino | 2004 | 0.5 (0.39–0.62) |
| Hispanic or Latino | 2005 | 0.49 (0.39–0.61) |
| Hispanic or Latino | 2006 | 0.73 (0.59–0.86) |
| Hispanic or Latino | 2007 | 0.58 (0.46–0.69) |
| Hispanic or Latino | 2008 | 0.68 (0.56–0.80) |
| Hispanic or Latino | 2009 | 0.76 (0.64–0.88) |
| Hispanic or Latino | 2010 | 0.75 (0.63–0.87) |
| Hispanic or Latino | 2011 | 0.78 (0.66–0.90) |
| Hispanic or Latino | 2012 | 0.87 (0.75–0.99) |
| Hispanic or Latino | 2013 | 1.03 (0.90–1.16) |
| Hispanic or Latino | 2014 | 0.81 (0.70–0.92) |
| Hispanic or Latino | 2015 | 0.96 (0.85–1.08) |
| Hispanic or Latino | 2016 | 1.01 (0.89–1.13) |
| Hispanic or Latino | 2017 | 1 (0.89–1.12) |
| Hispanic or Latino | 2018 | 1.02 (0.91–1.14) |
| Hispanic or Latino | 2019 | 0.95 (0.85–1.06) |
| Hispanic or Latino | 2020 | 1.64 (1.50–1.77) |
| Hispanic or Latino | 2021 | 1.48 (1.35–1.61) |
| Hispanic or Latino | 2022 | 1.32 (1.20–1.44) |
| Hispanic or Latino | 2023 | 0.9 (0.80–1.00) |
| NH Black or African American | 1999 | 0.98 (0.84–1.11) |
| NH Black or African American | 2000 | 0.94 (0.80–1.07) |
| NH Black or African American | 2001 | 0.93 (0.80–1.07) |
| NH Black or African American | 2002 | 0.98 (0.85–1.12) |
| NH Black or African American | 2003 | 1.14 (1.00–1.28) |
| NH Black or African American | 2004 | 1.32 (1.17–1.47) |
| NH Black or African American | 2005 | 1.31 (1.16–1.46) |
| NH Black or African American | 2006 | 1.36 (1.21–1.51) |
| NH Black or African American | 2007 | 1.39 (1.24–1.54) |
| NH Black or African American | 2008 | 1.3 (1.16–1.44) |
| NH Black or African American | 2009 | 1.76 (1.59–1.92) |
| NH Black or African American | 2010 | 1.81 (1.64–1.97) |
| NH Black or African American | 2011 | 1.7 (1.54–1.86) |
| NH Black or African American | 2012 | 2.05 (1.88–2.22) |
| NH Black or African American | 2013 | 1.85 (1.69–2.01) |
| NH Black or African American | 2014 | 1.93 (1.77–2.09) |
| NH Black or African American | 2015 | 2.23 (2.06–2.40) |
| NH Black or African American | 2016 | 2.22 (2.06–2.39) |
| NH Black or African American | 2017 | 2.35 (2.18–2.52) |
| NH Black or African American | 2018 | 2.53 (2.36–2.70) |
| NH Black or African American | 2019 | 2.4 (2.23–2.57) |
| NH Black or African American | 2020 | 3.98 (3.77–4.19) |
| NH Black or African American | 2021 | 4.15 (3.94–4.37) |
| NH Black or African American | 2022 | 3.43 (3.24–3.63) |
| NH Black or African American | 2023 | 2.89 (2.71–3.07) |
| NH White | 1999 | 0.82 (0.77–0.86) |
| NH White | 2000 | 0.86 (0.81–0.90) |
| NH White | 2001 | 0.92 (0.87–0.96) |
| NH White | 2002 | 1.01 (0.96–1.05) |
| NH White | 2003 | 1.16 (1.11–1.21) |
| NH White | 2004 | 1.21 (1.16–1.26) |
| NH White | 2005 | 1.34 (1.29–1.40) |
| NH White | 2006 | 1.42 (1.36–1.47) |
| NH White | 2007 | 1.45 (1.39–1.50) |
| NH White | 2008 | 1.55 (1.50–1.61) |
| NH White | 2009 | 1.65 (1.59–1.71) |
| NH White | 2010 | 1.75 (1.70–1.81) |
| NH White | 2011 | 1.92 (1.86–1.98) |
| NH White | 2012 | 1.99 (1.93–2.05) |
| NH White | 2013 | 2.2 (2.14–2.27) |
| NH White | 2014 | 2.24 (2.18–2.31) |
| NH White | 2015 | 2.35 (2.28–2.42) |
| NH White | 2016 | 2.43 (2.36–2.49) |
| NH White | 2017 | 2.5 (2.44–2.57) |
| NH White | 2018 | 2.54 (2.48–2.61) |
| NH White | 2019 | 2.55 (2.49–2.62) |
| NH White | 2020 | 3.43 (3.35–3.51) |
| NH White | 2021 | 4.04 (3.95–4.12) |
| NH White | 2022 | 3.57 (3.49–3.65) |
| NH White | 2023 | 3.11 (3.03–3.18) |

**Supplementary Table S2:** Overall Age Adjusted Mortality Rates (95% CI) stratified by ethnicity / race
