# Supplementary material for: Trends in mortality involving chronic obstructive pulmonary disease and obesity in the United States, 1999–2023: A population-based observational study
Source: Medicine (Baltimore). 2026 Oct 2;105(40):e50980. doi: 10.1097/MD.0000000000050980 (PMC13632968; doi:10.1097/MD.0000000000050980)
Supplement: Supplementary file 3 [file medi-105-e50980-s003.docx]

| Overall Age Adjusted Mortality Rate (95% CI) | | | |
| --- | --- | --- | --- |
| **Year** | **Young Adults** | **Middle-Aged Adults** | **Old Adults** |
| 1999 | 0.11 (0.09–0.13) | 1.17 (1.09–1.26) | 2.53 (2.36–2.69) |
| 2000 | 0.11 (0.09–0.13) | 1.19 (1.11–1.28) | 2.63 (2.46–2.80) |
| 2001 | 0.16 (0.13–0.19) | 1.31 (1.22–1.40) | 2.67 (2.50–2.84) |
| 2002 | 0.16 (0.13–0.19) | 1.41 (1.32–1.50) | 2.96 (2.78–3.14) |
| 2003 | 0.16 (0.14–0.19) | 1.61 (1.52–1.71) | 3.37 (3.18–3.56) |
| 2004 | 0.26 (0.23–0.30) | 1.71 (1.61–1.81) | 3.50 (3.30–3.69) |
| 2005 | 0.21 (0.18–0.24) | 1.87 (1.77–1.97) | 3.92 (3.72–4.12) |
| 2006 | 0.21 (0.18–0.24) | 1.89 (1.79–1.99) | 4.25 (4.04–4.46) |
| 2007 | 0.21 (0.18–0.24) | 2.03 (1.93–2.13) | 4.11 (3.91–4.32) |
| 2008 | 0.26 (0.23–0.30) | 2.03 (1.93–2.13) | 4.48 (4.27–4.70) |
| 2009 | 0.26 (0.23–0.30) | 2.33 (2.22–2.43) | 4.70 (4.49–4.91) |
| 2010 | 0.32 (0.28–0.36) | 2.37 (2.26–2.47) | 5.21 (4.98–5.43) |
| 2011 | 0.32 (0.28–0.36) | 2.47 (2.36–2.57) | 5.55 (5.32–5.78) |
| 2012 | 0.32 (0.28– 0.36) | 2.65 (2.54–2.76) | 5.86 (5.63–6.09) |
| 2013 | 0.37 (0.33–0.42) | 2.89 (2.77–3.00) | 6.21 (5.98–6.45) |
| 2014 | 0.32 (0.28–0.36) | 2.94 (2.83–3.06) | 6.35 (6.12–6.58) |
| 2015 | 0.32 (0.28–0.36) | 3.08 (2.97–3.20) | 6.73 (6.50–6.97) |
| 2016 | 0.32 (0.28–0.36) | 3.22 (3.10–3.34) | 6.97 (6.73–7.21) |
| 2017 | 0.32 (0.28–0.36) | 3.18 (3.06–3.30) | 7.41 (7.17–7.65) |
| 2018 | 0.32 (0.28–0.36) | 3.34 (3.22–3.46) | 7.47 (7.24–7.71) |
| 2019 | 0.26 (0.23–0.30) | 3.28 (3.16–3.40) | 7.45 (7.22–7.68) |
| 2020 | 0.37 (0.33–0.41) | 4.45 (4.31–4.59) | 10.62 (10.35–10.89) |
| 2021 | 0.48 (0.43–0.53) | 5.06 (4.92–5.21) | 11.95 (11.66–12.24) |
| 2022 | 0.37 (0.33–0.42) | 4.27 (4.13–4.40) | 10.92 (10.65–11.19) |
| 2023 | 0.32 (0.28–0.36) | 3.61 (3.49–3.74) | 9.23 (8.98–9.48) |

**Supplementary Table S3:** Overall Age Adjusted Mortality Rates (95% CI) stratified by age groups
