# Supplementary material for: Trends in mortality involving chronic obstructive pulmonary disease and obesity in the United States, 1999–2023: A population-based observational study
Source: Medicine (Baltimore). 2026 Oct 2;105(40):e50980. doi: 10.1097/MD.0000000000050980 (PMC13632968; doi:10.1097/MD.0000000000050980)
Supplement: Supplementary file 4 [file medi-105-e50980-s004.docx]

| Overall Age Adjusted Mortality Rate (95% CI) | | |
| --- | --- | --- |
| **Year** | **Metropolitan Regions** | **Non-Metropolitan Regions** |
| 1999 | 0.76(0.71–0.80) | 0.94 (0.84–1.04) |
| 2000 | 0.77(0.73–0.81) | 1.01 (0.91–1.11) |
| 2001 | 0.83(0.79–0.87) | 0.96 (0.86–1.05) |
| 2002 | 0.89(0.84–0.93) | 1.13 (1.02–1.23) |
| 2003 | 0.98(0.93–1.02) | 1.43 (1.31–1.54) |
| 2004 | 1.06(1.01–1.11) | 1.50 (1.38–1.62) |
| 2005 | 1.14(1.10–1.19) | 1.63 (1.51–1.75) |
| 2006 | 1.19(1.14–1.24) | 1.65 (1.52–1.77) |
| 2007 | 1.22(1.17–1.27) | 1.80 (1.67–1.93) |
| 2008 | 1.29(1.24–1.34) | 1.90 (1.77–2.03) |
| 2009 | 1.41(1.36–1.46) | 2.06 (1.93–2.20) |
| 2010 | 1.48(1.43–1.53) | 2.27 (2.13–2.41) |
| 2011 | 1.58(1.53–1.64) | 2.37 (2.22–2.51) |
| 2012 | 1.65(1.60–1.71) | 2.56 (2.41–2.71) |
| 2013 | 1.80(1.75–1.86) | 2.66 (2.50–2.81) |
| 2014 | 1.84(1.79–1.90) | 2.79 (2.63–2.94) |
| 2015 | 1.91(1.85–1.96) | 3.01 (2.85–3.17) |
| 2016 | 2.00(1.95–2.06) | 3.09 (2.93–3.25) |
| 2017 | 2.00(1.95–2.06) | 3.27 (3.11–3.43) |
| 2018 | 2.06(2.01–2.12) | 3.32 (3.15–3.48) |
| 2019 | 2.01(1.96–2.07) | 3.45 (3.28–3.62) |
| 2020 | 2.85(2.79–2.92) | 4.66 (4.47–4.86) |

**Supplementary Table S4:** Overall Age Adjusted Mortality Rates (95% CI) stratified by urbanization
