# Supplementary material for: Trends in mortality involving chronic obstructive pulmonary disease and obesity in the United States, 1999–2023: A population-based observational study
Source: Medicine (Baltimore). 2026 Oct 2;105(40):e50980. doi: 10.1097/MD.0000000000050980 (PMC13632968; doi:10.1097/MD.0000000000050980)
Supplement: Supplementary file 5 [file medi-105-e50980-s005.docx]

| Overall Age Adjusted Mortality Rate (95% CI) | | | | |
| --- | --- | --- | --- | --- |
| **Year** | **Census Region 1:**  **Northeast** | **Census Region 2:**  **Midwest** | **Census Region 3:**  **South** | **Census Region 4:**  **West** |
| 1999 | 0.70 (0.62–0.77) | 0.85 (0.77–0.93) | 0.72 (0.66–0.78) | 0.92 (0.83–1.01) |
| 2000 | 0.73 (0.65–0.81) | 0.87 (0.79–0.95) | 0.74 (0.68–0.80) | 0.99 (0.89–1.08) |
| 2001 | 0.75 (0.67–0.83) | 0.93 (0.85–1.01) | 0.77 (0.71–0.83) | 0.99 (0.90–1.08) |
| 2002 | 0.80 (0.72–0.88) | 0.97 (0.88–1.05) | 0.88 (0.82–0.95) | 1.07 (0.98–1.17) |
| 2003 | 0.92 (0.84–1.01) | 1.20 (1.11–1.30) | 1.05 (0.98–1.12) | 1.09 (0.99–1.18) |
| 2004 | 0.90 (0.81–0.99) | 1.29 (1.20–1.39) | 1.03 (0.96–1.10) | 1.33 (1.22–1.43) |
| 2005 | 0.97 (0.88–1.06) | 1.49 (1.39–1.59) | 1.20 (1.13–1.27) | 1.25 (1.15–1.34) |
| 2006 | 1.10 (1.01–1.20) | 1.42 (1.32–1.52) | 1.26 (1.18–1.33) | 1.41 (1.31–1.51) |
| 2007 | 1.18 (1.08–1.27) | 1.41 (1.32–1.51) | 1.22 (1.15–1.30) | 1.48 (1.38–1.59) |
| 2008 | 1.16 (1.06–1.26) | 1.60 (1.49–1.70) | 1.34 (1.27–1.41) | 1.46 (1.36–1.56) |
| 2009 | 1.24 (1.14–1.34) | 1.78 (1.67–1.89) | 1.49 (1.41–1.57) | 1.56 (1.46–1.66) |
| 2010 | 1.36 (1.26–1.46) | 1.78 (1.67–1.89) | 1.62 (1.54–1.70) | 1.65 (1.54–1.75) |
| 2011 | 1.50 (1.39–1.61) | 2.03 (1.92–2.15) | 1.59 (1.51–1.67) | 1.81 (1.70–1.92) |
| 2012 | 1.58 (1.47–1.69) | 2.12 (2.00–2.23) | 1.80 (1.72–1.89) | 1.68 (1.58–1.78) |
| 2013 | 1.53 (1.42–1.63) | 2.27 (2.15–2.39) | 1.96 (1.87–2.05) | 1.97 (1.86–2.08) |
| 2014 | 1.58 (1.47–1.69) | 2.37 (2.25–2.50) | 2.00 (1.92–2.09) | 1.88 (1.77–1.99) |
| 2015 | 1.64 (1.53–1.75) | 2.49 (2.37–2.61) | 2.07 (1.98–2.15) | 2.07 (1.96–2.18) |
| 2016 | 1.81 (1.69–1.92) | 2.54 (2.41–2.66) | 2.09 (2.00–2.17) | 2.19 (2.08–2.30) |
| 2017 | 1.87 (1.76–1.99) | 2.75 (2.62–2.88) | 2.18 (2.09–2.26) | 2.07 (1.97–2.18) |
| 2018 | 1.84 (1.72–1.95) | 2.65 (2.53–2.78) | 2.26 (2.17–2.34) | 2.23 (2.12–2.34) |
| 2019 | 1.85 (1.74–1.96) | 2.54 (2.41–2.66) | 2.32 (2.24–2.41) | 2.13 (2.02–2.24) |
| 2020 | 2.60 (2.46–2.73) | 3.76 (3.61–3.91) | 3.26 (3.15–3.36) | 2.71 (2.59–2.83) |
| 2021 | 2.57 (2.43–2.70) | 4.08 (3.93–4.24) | 3.86 (3.74–3.97) | 3.24 (3.11–3.37) |
| 2022 | 2.42 (2.30–2.55) | 3.39 (3.25–3.53) | 3.43 (3.33–3.54) | 2.86 (2.74–2.98) |
| 2023 | 1.99 (1.88–2.11) | 2.82 (2.69–2.95) | 3.00 (2.90–3.09) | 2.30 (2.20–2.41) |

**Supplementary Table S5:** Overall Age Adjusted Mortality Rates (95% CI) stratified by Census Regions
