# Supplementary material for: Trends in mortality involving chronic obstructive pulmonary disease and obesity in the United States, 1999–2023: A population-based observational study
Source: Medicine (Baltimore). 2026 Oct 2;105(40):e50980. doi: 10.1097/MD.0000000000050980 (PMC13632968; doi:10.1097/MD.0000000000050980)
Supplement: Supplementary file 6 [file medi-105-e50980-s006.docx]

| **State** | **AAMR (95% CI), 1999–2020** | **AAMR (95% CI), 2021–2023** |
| --- | --- | --- |
| Alabama | 1.31 (1.23–1.39) | 2.91 (2.62–3.24) |
| Alaska | 2.31 (1.95–2.66) | 2.47 (1.78–3.43) |
| Arizona | 1.52 (1.44–1.60) | 3.31 (3.05–3.59) |
| Arkansas | 1.83 (1.71–1.95) | 4.54 (4.05–5.07) |
| California | 1.86 (1.83–1.90) | 2.39 (2.29–2.49) |
| Colorado | 3.19 (3.06–3.33) | 6.12 (5.70–6.56) |
| Connecticut | 1.23 (1.14–1.32) | 1.72 (1.46–2.02) |
| Delaware | 2.53 (2.28–2.79) | 6.25 (5.36–7.28) |
| District of Columbia | 1.79 (1.51–2.07) | 2.52 (1.74–3.56) |
| Florida | 1.71 (1.67–1.75) | 2.87 (2.74–3.01) |
| Georgia | 1.63 (1.56–1.70) | 4.09 (3.84–4.36) |
| Hawaii | 1.04 (0.90–1.17) | 1.45 (1.10–1.90) |
| Idaho | 2.37 (2.17–2.57) | 5.68 (5.01–6.44) |
| Illinois | 1.53 (1.47–1.58) | 2.57 (2.40–2.76) |
| Indiana | 2.91 (2.80–3.01) | 4.47 (4.15–4.81) |
| Iowa | 3.25 (3.09–3.41) | 6.00 (5.46–6.59) |
| Kansas | 2.31 (2.16–2.45) | 4.68 (4.18–5.24) |
| Kentucky | 2.91 (2.78–3.04) | 5.84 (5.39–6.33) |
| Louisiana | 1.77 (1.67–1.87) | 4.36 (3.96–4.78) |
| Maine | 2.36 (2.17–2.55) | 3.62 (3.03–4.32) |
| Maryland | 1.67 (1.59–1.76) | 3.57 (3.27–3.89) |
| Massachusetts | 1.20 (1.14–1.27) | 1.85 (1.65–2.07) |
| Michigan | 2.22 (2.15–2.30) | 3.79 (3.55–4.04) |
| Minnesota | 2.39 (2.28–2.49) | 4.90 (4.53–5.28) |
| Mississippi | 2.29 (2.14–2.43) | 4.90 (4.38–5.46) |
| Missouri | 1.99 (1.90–2.08) | 3.29 (3.00–3.60) |
| Montana | 3.02 (2.76–3.28) | 5.40 (4.59–6.34) |
| Nebraska | 2.48 (2.30–2.67) | 4.73 (4.12–5.42) |
| Nevada | 1.94 (1.80–2.08) | 4.16 (3.71–4.65) |
| New Hampshire | 2.20 (2.00–2.39) | 3.63 (3.04–4.32) |
| New Jersey | 1.54 (1.47–1.60) | 1.74 (1.57–1.92) |
| New Mexico | 2.05 (1.89–2.21) | 3.31 (2.84–3.85) |
| New York | 1.49 (1.45–1.54) | 2.57 (2.43–2.72) |
| North Carolina | 2.34 (2.26–2.41) | 3.12 (2.91–3.34) |
| North Dakota | 2.64 (2.33–2.95) | 5.25 (4.22–6.49) |
| Ohio | 2.64 (2.57–2.72) | 4.11 (3.88–4.35) |
| Oklahoma | 4.39 (4.22–4.56) | 13.63 (12.88–14.42) |
| Oregon | 2.82 (2.69–2.95) | 5.08 (4.67–5.53) |
| Pennsylvania | 1.92 (1.86–1.98) | 3.67 (3.46–3.88) |
| Rhode Island | 2.87 (2.62–3.12) | 8.09 (7.08–9.24) |
| South Carolina | 2.31 (2.20–2.42) | 9.72 (9.20–10.27) |
| South Dakota | 2.30 (2.04–2.56) | 5.56 (4.59–6.70) |
| Tennessee | 2.71 (2.60–2.81) | 5.15 (4.81–5.51) |
| Texas | 2.03 (1.98–2.08) | 3.27 (3.12–3.41) |
| Utah | 1.30 (1.17–1.42) | 2.97 (2.56–3.44) |
| Vermont | 6.87 (6.38–7.37) | 11.75 (10.18–13.56) |
| Virginia | 1.15 (1.09–1.21) | 2.14 (1.95–2.35) |
| Washington | 2.57 (2.47–2.67) | 4.58 (4.28–4.90) |
| West Virginia | 3.45 (3.24–3.66) | 7.32 (6.53–8.20) |
| Wisconsin | 2.84 (2.73–2.95) | 6.45 (6.05–6.88) |
| Wyoming | 4.11 (3.68–4.54) | 8.97 (7.48–10.73) |

**Supplementary Table S7:** Total Age Adjusted Mortality Rate stratified by States
