# Supplementary material for: Trends in mortality involving chronic obstructive pulmonary disease and obesity in the United States, 1999–2023: A population-based observational study
Source: Medicine (Baltimore). 2026 Oct 2;105(40):e50980. doi: 10.1097/MD.0000000000050980 (PMC13632968; doi:10.1097/MD.0000000000050980)
Supplement: Supplementary file 7 [file medi-105-e50980-s007.docx]

| Place of Death | Deaths | Percentage of Deaths (%) |
| --- | --- | --- |
| Medical Facility - Inpatient | 44237 | 33.78 |
| Medical Facility - Outpatient or ER | 14446 | 11.03 |
| Medical Facility - Dead on Arrival | 1136 | 0.87 |
| Medical Facility - Status unknown | 37 | 0.03 |
| Decedent's home | 48971 | 37.39 |
| Hospice facility | 3123 | 2.38 |
| Nursing home/long term care | 13899 | 10.61 |
| Other | 4927 | 3.76 |
| Place of death unknown | 182 | 0.14 |

**Supplementary Table S6:** Total deaths and percentage of deaths stratified by Place of Death

*Note: Percentages were calculated using the full analytic cohort of 130,958 deaths and are reported to two decimal places. Displayed percentages sum to 99.99% and may not total exactly 100.00% due to rounding.*
